# 

**Published:** 1886-08

**Authors:** 


CONTENTS:

ARTICLE I—The Preparation of the Mouth for and the Insertion of Artificial Teeth. By John Trude Fripp, L. D. S., Edin. & I. .... 145

" II—Prosthetic Dentistry. By James B Hodgkin, D. D. S. .... 157

" III—Odontological Society of Pennsylvania .... 162

" IV—National Association of Dental Faculties .... 175

" V—Care and Treatment of Children's Teeth. By L. S. Chilcott, D. D. S. .. 179

EDITORIAL, ETC.

The Southern Dental Association .... 183

American Dental Association .... 185

MONTHLY SUMMARY.

A Disagreeable Accident Connected with the Extraction of a Tooth .... 187
All Porcelain Crowns and Bridge Work .... 188
The Destructive Energy of the Tincture of the Chloride of Iron on the Teeth .... 190

